# Exceptionally prolonged tooth formation in elasmosaurid plesiosaurians

**DOI:** 10.1371/journal.pone.0172759

**Published:** 2017-02-27

**Authors:** Benjamin P. Kear, Dennis Larsson, Johan Lindgren, Martin Kundrát

**Affiliations:** 1 Museum of Evolution, Uppsala University, Uppsala, Sweden; 2 Department of Organismal Biology, Uppsala University, Uppsala, Sweden; 3 Department of Geology, Lund University, Lund, Sweden; 4 Center for Interdisciplinary Biosciences, Faculty of Science, University of Pavol Jozef Safarik, Jesenna 5, SK Kosice, Slovak Republic; University of Michigan, UNITED STATES

## Abstract

Elasmosaurid plesiosaurians were globally prolific marine reptiles that dominated the Mesozoic seas for over 70 million years. Their iconic body-plan incorporated an exceedingly long neck and small skull equipped with prominent intermeshing ‘fangs’. How this bizarre dental apparatus was employed in feeding is uncertain, but fossilized gut contents indicate a diverse diet of small pelagic vertebrates, cephalopods and epifaunal benthos. Here we report the first plesiosaurian tooth formation rates as a mechanism for servicing the functional dentition. Multiple dentine thin sections were taken through isolated elasmosaurid teeth from the Upper Cretaceous of Sweden. These specimens revealed an average of 950 daily incremental lines of von Ebner, and infer a remarkably protracted tooth formation cycle of about 2–3 years–other polyphyodont amniotes normally take ~1–2 years to form their teeth. Such delayed odontogenesis might reflect differences in crown length and function within an originally uneven tooth array. Indeed, slower replacement periodicity has been found to distinguish larger caniniform teeth in macrophagous pliosaurid plesiosaurians. However, the archetypal sauropterygian dental replacement system likely also imposed constraints via segregation of the developing tooth germs within discrete bony crypts; these partly resorbed to allow maturation of the replacement teeth within the primary alveoli after displacement of the functional crowns. Prolonged dental formation has otherwise been linked to tooth robustness and adaption for vigorous food processing. Conversely, elasmosaurids possessed narrow crowns with an elongate profile that denotes structural fragility. Their apparent predilection for easily subdued prey could thus have minimized this potential for damage, and was perhaps coupled with selective feeding strategies that ecologically optimized elasmosaurids towards more delicate middle trophic level aquatic predation.

## Introduction

**Fig 1:**
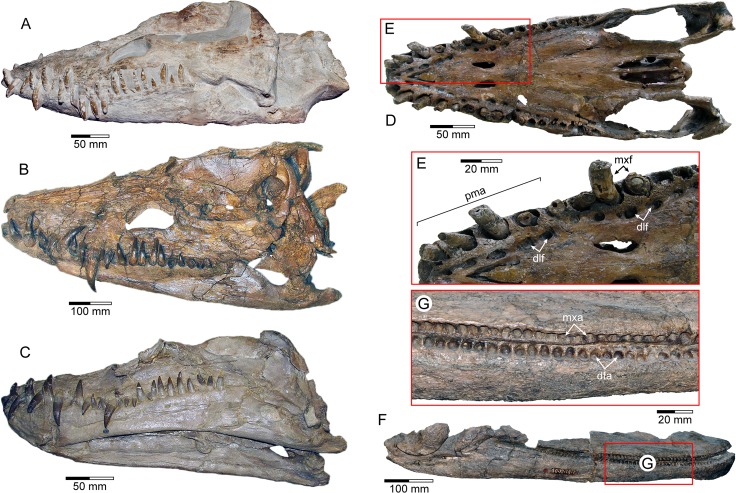
Comparative dental arrangements in elasmosaurid plesiosaurians. Crania and mandibles of (A) *Libonectes atlasense*, Staatliches Museum für Naturkunde Karlsruhe (SMNK) PAL 3978, Germany; (B) *Thalassomedon hanningtoni*, University of Nebraska State Museum (UNSM) 50132, U.S.A.; (C) *Styxosaurus snowii*, Kansas University Museum of Natural History (KUVP) 1301, U.S.A.; (D) *Libonectes morgani*, Southern Methodist University Schuler Museum of Paleontology (SMUSMP) 691, U.S.A., in palatal view with (E) enlargement of the premaxillary rostrum; (F) *Aristonectes parvidens*, Museo La Plata (MLP) 40-XI-14-6, Argentina in lateral view with (G) enlargement of the maxillary and dentary alveoli. Scales = 50 mm in (A, C, D); 20 mm in (E, G); 100 mm in (B, F). Abbreviations: dta, dentary alveoli; dlf, dental lamina foramina; pma, premaxillary alveoli; mxa, maxillary alveoli; mxf, enlarged maxillary ‘fangs’.

Plesiosaurians (Plesiosauria) were highly diverse Mesozoic marine amniotes whose fossil record extended over 135 million years. During this vast timeframe the clade achieved a variety of body forms and feeding modes ranging from massive-skulled megacarnivores (e.g. the famous pliosaurid *Liopleurodon* [1]), apparently specialized for enormous bite forces and hydrodynamic agility [2–5], to small-prey specialists epitomized by the Elasmosauridae, whose immensely long necks, typically diminutive heads (*ca* 330 mm versus an eight metre maximum body length in *Hydrotherosaurus* [6]) and meshwork of slender ‘fang-like’ teeth (Fig 1A–1C) constitute one of the most extreme adaptive morphologies yet evidenced amongst aquatic vertebrates [7]. The functionality of this bizarre feeding system has long been contested with contrasting hypotheses advocating ‘swan-like’ fishing with the head craned above the water [8], to ambush hunting of pelagic prey [8–11], and use of the teeth to aggressively stun [12], passively strain [13], or ‘graze’ along the sea floor [2, 14]. Irrespectively, structural modeling has inferred both a limited capacity for neck movement [12], and optimization of the cranial architecture towards rapid jaw closure [15]. In addition, direct evidence from preserved gut contents indicates an assorted diet of bivalves, gastropods and crinoids [14], as well as small bony fish, ammonites, pterosaurs and perhaps even juvenile mosasaurs (although the latter dietary association has been questioned [10, 16]). How this spectrum of prey was captured and processed is undetermined, but the characteristic dentition of elasmosaurids presumably played a primary role.

The teeth of elasmosaurids were distinctive amongst plesiosaurians in their elongate tapered profile and often labiolingually-compressed crowns (imparting an oval to elliptical cross-section [17]) that were ornamented by numerous fine enamel ridges. The dentition was typically anisodont (= incorporating regionalized size variation, but differing from heterodonty, in which tooth shape is substantially modified: *sensu* [18]) with diastemata interspersed between the inclined premaxillary and symphyseal dentary alveoli (Fig 1D and 1E). Vertical caniniform ‘fangs’ (*ca* 45/10 mm in maximum crown height/basal diameter [19]) were also situated at the premaxillary-maxillary suture, and along the dentary, but these reduced in length towards the rear of the jaw (Fig 1A–1E). Some derived elasmosaurid taxa (e.g. *Aristonectes* [13, 20, 21]) alternatively manifested densely packed alveoli that were more evenly spaced (Fig 1F and 1G); this implies a homodont (= equally sized: *ca* 20 mm in crown height [17]) dental array, but with basic tooth shape and intercalation [13, 20] similar to other elasmosaurids including anisodont basal forms [19, 22–24].

**Fig 2:**
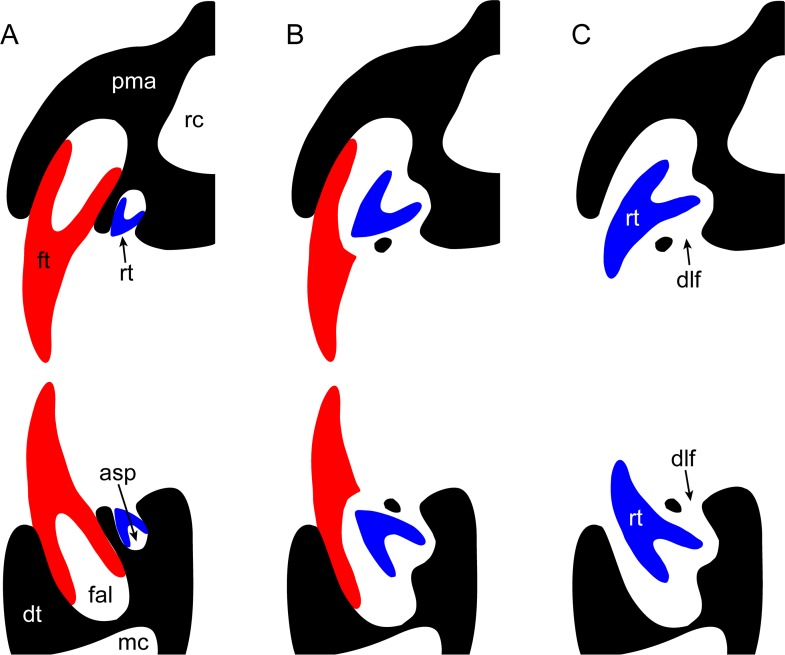
Simplified diagram of plesiosaurian tooth replacement (adapted from Sassoon et al.[18]). Sequence depicts: (A) formation of the replacement tooth within the ‘alveolarized’ bony crypt; (B) resorbtion of the dental lamina; and (C) maturation of the replacement tooth within the primary alveolus after displacement of the functional crown. Abbreviations: asp, alveolar space; dlf, dental lamina foramina; dt, dentary; fal, functional alveolus; ft, functional tooth; pma, premaxilla; mc, medullary canal; rc, rostral cavity; rt, replacement tooth.

Like other plesiosaurians [18, 25], and Triassic sauropterygians [25–28], elasmosaurids were polyphyodont (= undergoing continuous tooth shedding and replacement throughout life) with a unique dental development cycle (Fig 2A–2C) involving formation of isolated tooth germs within discrete bony crypts (= alveolar spaces [18]). As these grew, the emergent crowns exposed through dental lamina foramina perforating the dentigerous bone medial to the functional tooth row [18, 26]. The labial wall of the ‘alveolarized’ [26] crypt subsequently resorbed, and the functional tooth was shed while its replacement migrated into the primary alveolus. Maturation was thus completed prior to final eruption, which took place either in symmetrical pairs coordinated across the midline of the jaws (e.g. anisodont plesiosaurians), or asymmetrically in the rear-most teeth of some heterodont pliosaurids (e.g. *Pliosaurus* [18]). The precise rate at which this process occurred is unknown, but has critical implications for the mode of prey capture since incremental deposition of dentine and enamel occurs independent of functional tooth breakage, wear or loss [29]. Therefore, developmental duration and pattern can inhibit optimal operation of the dentition through increased risk of damage during feeding [30].

## Materials and methods

**Fig 3:**
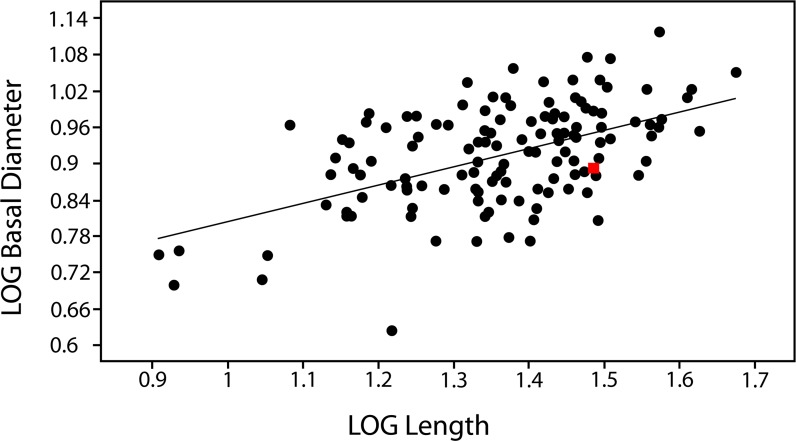
Bivariate plot of tooth proportions in the PMU *Scanisaurus* prototype dentition. Visualization of log_10_-transformed data generated in *PAST* [59] implied a consistent linear relationship between maximum base/height proportions within the sample (RMA slope *a*   =   0.30419, *r*   =   0.53022). The most intact available tooth (PMU 28757: red square) was used to calculate tooth formation time.

Because complete *in situ* elsmosaurid dentitions are exceptionally rare and not readily accessible for destructive analysis, we obtained a discrete sample of 131 isolated elasmosaurid teeth, historically referred to *Scanisaurus* [31–34], to reconstruct a prototype dental array. These specimens derived from the Museum of Evolution palaeontological collection (PMU) at Uppsala University in Sweden (Table 1), and were recovered from a stratigraphically restricted horizon of the uppermost lower Campanian (Late Cretaceous: ~80 mya) *Belemnellocamax mammillatus* belemnite zone at Åsen and Ivö Klack–two geographically proximal localities (~ 6 km apart) in the northeastern Kristianstad Basin of Skåne in southern Sweden [34, 35]. Almost all of the PMU *Scanisaurus* teeth exhibited damage from pre-burial abrasion, surface weathering and/or excavation, which precluded volumetric assessments of mean size based on complete teeth [30, 36, 37]. However, correlation between the maximum crown length/basal diameter dimensions in our sample (Fig 3) suggested consistent linear proportions despite breakage. The tooth size range (PMU 24468 at 8.1/5.6 mm, to PMU 24533 at 47.3/11.2 mm) also closely matched those recovered from *in situ* elasmosaurid dentitions (e.g. premaxilla/maxilla 10–45/5–10 mm; dentary 25–30/~7 mm [19]).

**Table 1:** Maximum preserved tooth height and mesial-distal diameter measurements (mm) of the PMU *Scanisaurus* prototype dentition.

Specimen	Height	Diam.	Specimen	Height	Diam.	Specimen	Height	Diam.
PMU 24421	28.8	8	PMU 24465	17.3	7.3	PMU 24510	31.3	9.1
PMU 24422	23.7	9.9	PMU 24466	20.5	7.6	PMU 24511	21.4	5.9
PMU 24423	15.5	8	PMU 24467	17.9	8.8	PMU 24512	25.2	5.9
PMU 24424	25.6	8.3	PMU 24468	8.1	5.6	PMU 24513	31.2	10.9
PMU 24425	22.7	8.5	PMU 24469	17.8	9.5	PMU 24514	18.1	7.3
PMU 24426	16.5	7.3	PMU 24470	29.5	10	PMU 24515	23.1	7.7
PMU 24427	29	10.2	PMU 24471	12.1	9.2	PMU 24516	26.6	7.1
PMU 24428	19.4	7.2	PMU 24473	26.7	10	PMU 24517	25.5	6.4
PMU 24429	14.7	7.8	PMU 24474	21.9	9	PMU 24518	21.2	7.7
PMU 24430	22	9.7	PMU 24475	15.4	9.6	PMU 24519	28.3	7.2
PMU 24431	28.7	10.9	PMU 24476	14.5	8.6	PMU 24520	27.3	8
PMU 24432	22.4	7.4	PMU 24477	14.4	6.6	PMU 24521	20.5	9.9
PMU 24433	25.3	9.3	PMU 24478	13.9	8.1	PMU 24522	28.1	8.3
PMU 24434	30	11.9	PMU 24479	13.5	6.8	PMU 24524	22.5	7.4
PMU 24435	18.9	9.2	PMU 24480	20.9	8.4	PMU 24525	24.6	8.7
PMU 24436	27.5	8.7	PMU 24481	14.2	8.7	PMU 24526	23.6	6
PMU 24437	19.6	9.2	PMU 24482	8.5	5	PMU 24527	17.3	9.5
PMU 24438	25.1	8.3	PMU 24483	25.7	6.7	PMU 24528	27.4	8.9
PMU 24439	26.3	10.8	PMU 24484	22	8.6	PMU 24533	47.3	11.2
PMU 24440	15.3	9.3	PMU 24485	23.9	11.4	PMU 24534	36	10.5
PMU 24441	16.2	9.1	PMU 24486	14.4	6.5	PMU 24535	36.4	9.2
PMU 24442	14.6	6.5	PMU 24487	17.5	6.5	PMU 24536	35.1	7.6
PMU 24443	21.4	8	PMU 24488	17.2	7.5	PMU 24537	37.7	9.4
PMU 24444	31.4	9.6	PMU 24489	13.7	7.6	PMU 24538	28.7	8.8
PMU 24445	20.8	10.8	PMU 24490	15	7.6	PMU 24539	40.8	10.2
PMU 24446	23.4	10.2	PMU 24491	17.3	7.2	PMU 24540	31.9	10.6
PMU 24447	27	9.4	PMU 24492	23.3	7.9	PMU 24541	37.3	9.1
PMU 24448	23	9.4	PMU 24493	31	6.4	PMU 24542	30.6	9.7
PMU 24449	22.8	7.6	PMU 24494	28	8.9	PMU 24543	32.2	8.7
PMU 24450	17.6	8.5	PMU 24495	23.1	6.9	PMU 24544	34.7	9.3
PMU 24451	21.5	8.6	PMU 24496	27.4	8	PMU 24545	22	6.5
PMU 24452	17.6	6.7	PMU 24497	27.2	9.6	PMU 24546	41.3	10.5
PMU 24453	16.5	4.2	PMU 24498	29.9	9.8	PMU 24547	42.3	9
PMU 24454	29.1	8.8	PMU 24499	26	8.9	PMU 24548	31.1	8.1
PMU 24455	26.4	9.5	PMU 24500	22.2	6.6	PMU 24549	31.2	8.6
PMU 24456	27.9	9.5	PMU 24501	36.5	8.8	PMU 28757	30.6	7.8
PMU 24457	18.9	5.9	PMU 24502	27.1	7.5	PMU 28758	21.5	6.9
PMU 24458	15.1	7	PMU 24503	22.5	10.2	PMU 28759	11.1	5.1
PMU 24459	11.3	5.6	PMU 24504	28.9	7.6	PMU 28760	8.6	5.7
PMU 24460	22.3	8.9	PMU 24505	30	7.1	PMU 28761	30.8	7.6
PMU 24461	37.4	13.1	PMU 24506	21.4	7.2	PMU 28762	29	9.1
PMU 24462	35.9	8	PMU 24507	29.7	7.7	PMU 28763	32.2	11.8
PMU 24463	21.5	7.1	PMU 24508	23.4	7.4	**Mean**	**24.2**	**8.3**
PMU 24464	25.8	7.2	PMU 24509	24.4	6.9	**SD**	**7.5**	**1.5**

**Fig 4:**
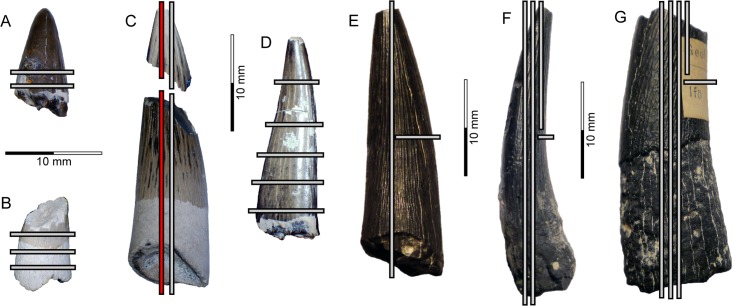
Pre-sectioned PMU *Scanisaurus* teeth. Crowns in (A–E) lingual, and (F, G) mesio-distal views. Section sub-numbering is apical–basal, and left–right: (A) PMU 28759/1–2; (B) PMU 28760/1–3; (C) PMU 28757/1–4 (longitudinal midline section highlighted in red); (D) PMU 28758/1–5; (E) PMU 28762/1–2; (F) PMU 28761/1–4; (G) PMU 28763/1–5. Scale = 10 mm in (A–G).

**Fig 5:**
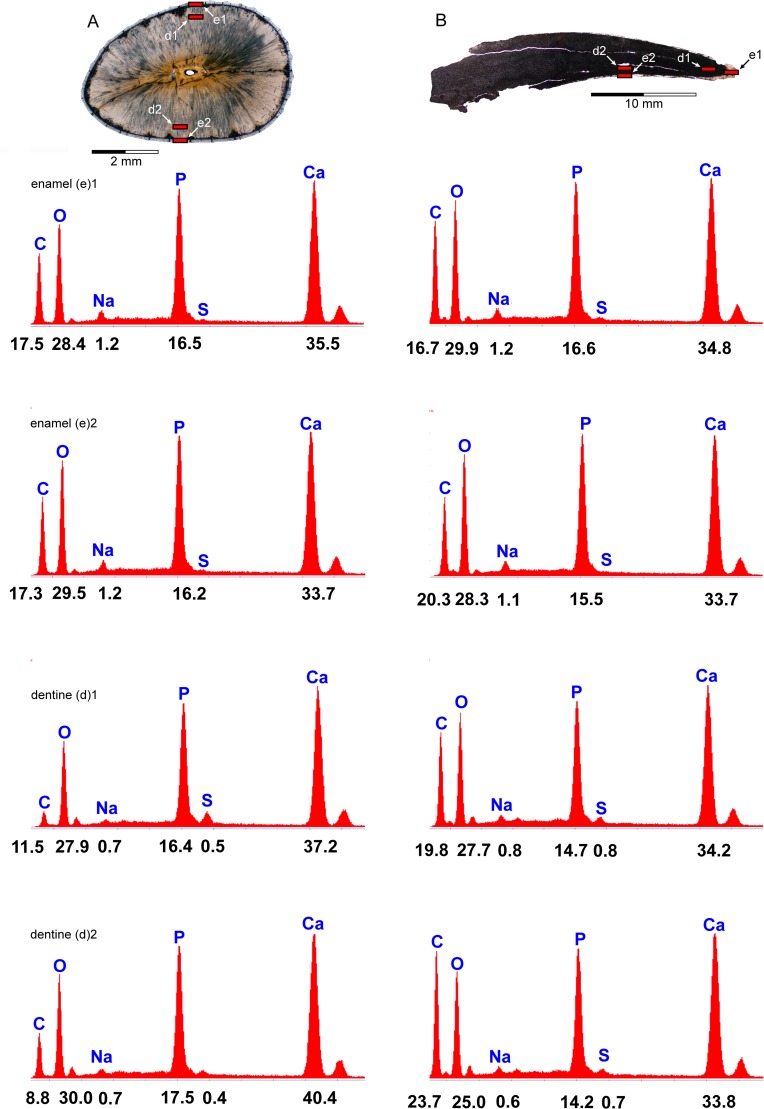
EDX spectra from selected PMU *Scanisaurus* teeth. These indicate compositional proportions (% weight) of primary elements in (A) PMU 28758/4, and (B) PMU 28761/1.

**Fig 6:**
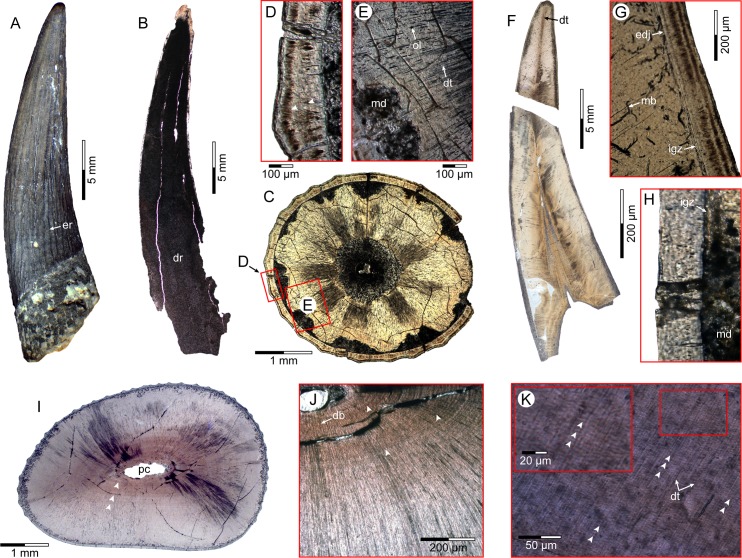
PMU *Scanisaurus* tooth sections. (A) PMU 24517; (B) PMU 28761/1; (C) PMU 28759/1; (D) PMU 28759/1 enamel laminae (arrows); (E) PMU 28759/1 dentine microstructures; (F) PMU 28757/1–2; (G) and (H) PMU 28757/4 enlargements of enamel-dentine junction; (I) PMU 28758/5 discoloured dentine bands (arrows); (J) PMU 28758/4 enlargement of possible long-period increments (arrows); (K) PMU 28757/2 short period increments (arrows). Abbreviations: db, discoloured dentine band; dr, diagenetic recrystalization; dt, dentinal tubules; edj, enamel-dentine junction; er, enamel ridge; igz, interglobular zone; md, microstructural degradation; ol, osteocyte lacunae; mb, microboring; pc, pulp cavity.

Four teeth from Åsen (PMU 28757–PMU 28760), and three from Ivö Klack (PMU 28761–PMU 28763) were selected for thin sectioning based on overall crown completeness and/or intact roots. These specimens were embedded in bicomponent epoxy resin (*Lamit* 109, *Kittfort*) and impregnated with *EpoFix* (*Struers*) prior to cutting on a 150 mm diamond blade, and abrasion to 0.2 mm on a Montasupal grinder (grits: 240, 400, 600) using silicon carbide. The resulting 25 mounted petrographic slides (Fig 4) were inspected on a *Leitz* DM RXE microscope equipped with a *Leica* DFC 550 camera and *LAS* v.4.2 software. Diagenetic modification was assessed using Field emission gun scanning electron microscopy (FEG-SEM) undertaken on uncoated slides with a *Zeiss* Supra 35-VP (*Carl Zeiss SMT*) incorporating a low vacuum VPSE detector, Robinson BSD backscattered electron-imaging attachment, and coupled EDX Apex 4 (*Ametekh*) EDS-detector for dispersive X-ray microanalysis. This detected high component phosphate, implying minimal elemental alteration in the samples from Åsen (Fig 5). Extensive mineral recrystalization otherwise entirely obscured dentine microstructures in the teeth from Ivö Klack (Fig 6A and 6B) rendering them unsuitable for further examination.

In accordance with previous studies [30, 36, 37], we used a continuous longitudinal midline section through the apex (PMU 28757/1) and crown base (PMU 28757/2) of the most complete available tooth (proportionately sized within one standard deviation of the sample mean: Fig 3; Table 1) to calculate tooth formation time. However, other longitudinal (PMU 28757/3, PMU 28757/4) and transverse sections were made through additional teeth (PMU 28759/1–2, PMU 28758/1–5) to inspect enamel/dentine microstructures in different aspects. Dentine deposition rates were based on counts of short-period incremental growth lines of von Ebner [30, 36–38], which are laid down following a circadian biorhythm in all tooth-bearing vertebrates [30, 36–42]. Nevertheless, the identification of such markings has differed between recent studies [37, 39], with Dean [40–42] providing an explicit characterization of von Ebner lines as narrowly-spaced fine layers <3 μm apart, and distinct from coarser Andresen lines, which can be up to 20 μm and represent longer-cycle periodicity. This pattern of two types of interpolating increments was visible in our fossils, therefore we proceeded by taking measurements based on high-resolution digital micrographs in *ImageJ* [43, 44]. Sequential sets of 10 clearly visible short-period lines (Table 2) were demarcated by the leading edge of one light-coloured increment band to the next, and measured at both labial and lingual locations perpendicular to the enamel-dentine junction, as well as from different locations closer to the cuspal apex (where dentine formation rates are known to increase [40]) and below the mid-length of the crown. Distal and medial locations were also examined in transverse sections to cross-reference results. A cumulative average of all short-period increment measurements was then multiplied by the mean dentine thickness taken across each location (Table 3); this established an estimate of the total dentine accumulation rate for each individual tooth [38].

**Table 2:** Measurements (μm) of short-period lines of von Ebner observed in the sectioned PMU *Scanisaurus* teeth. Abbreviations: LCA = longitudinal crown apex; LCMB = longitudinal mid-crown to base; TCA = transverse crown apex; TMC = transverse mid-crown.

Specimen	Section	Labial		Lingual		Mesial-Distal	
PMU 28757/1	LCA	3.6	2.7	1.4	2	-	-
		3.7	3	1.5	2.1	-	-
		3.7	3.5	1.6	2.1	-	-
		3.9	3.9	1.9	2.2	-	-
		4	4.1	2.1	2.3	-	-
		4.4	4.1	2.4	2.3	-	-
		4.4	4.2	2.5	2.5	-	-
		4.9	4.3	2.7	2.6	-	-
		5	4.7	2.8	2.6	-	-
		5.3	5.1	2.9	2.8	-	-
	**Mean**	**4.29**	**3.96**	**2.18**	**2.35**	**-**	**-**
PMU 28757/2	LCMB	0.9	1.4	1.7	1.7	-	-
		1.2	1.5	1.7	1.8	-	-
		1.2	1.6	1.8	1.8	-	-
		1.3	1.7	1.9	1.8	-	-
		1.3	1.8	1.9	1.9	-	-
		1.3	1.9	2	2	-	-
		1.3	2.1	2.1	2.2	-	-
		1.4	2.2	2.1	2.3	-	-
		2	2.3	2.3	2.5	-	-
		2.1	2.3	2.3	2.7	-	-
	**Mean**	**1.4**	**1.88**	**1.98**	**2.07**	**-**	**-**
PMU 28759/1	TCA	2.7	-	2	-	1.3	1.5
		3	-	2.3	-	1.8	2
		3	-	2.3	-	1.8	2.1
		3.1	-	2.6	-	1.9	2.2
		3.1	-	2.6	-	2.4	2.6
		3.2	-	2.7	-	2.4	2.7
		3.4	-	2.8	-	2.5	2.8
		3.5	-	3.1	-	2.6	2.9
		3.7	-	3.1	-	2.8	3
		4	-	3.2	-	3.2	3.3
	**Mean**	**3.27**	**-**	**2.67**	**-**	**2.27**	**2.51**
PMU 28758/3	TMC	1.4	-	1.7	-	1.3	1.2
		1.5	-	2.3	-	1.5	1.2
		1.6	-	2.4	-	1.6	1.3
		1.7	-	2.4	-	1.7	1.3
		1.8	-	2.4	-	1.7	1.4
		1.8	-	2.5	-	1.8	1.5
		2	-	2.5	-	1.9	1.5
		2.1	-	2.5	-	1.9	1.7
		2.1	-	2.6	-	2	1.7
		2.1	-	3.1	-	2	1.9
	**Mean**	**1.81**	**-**	**2.44**	**-**	**1.74**	**1.47**

**Table 3:** Dentine width measurements (mm) from selected PMU *Scanisaurus* teeth. Abbreviations: LCA = longitudinal crown apex; LCMB = longitudinal mid-crown to base; LWT = longitudinal whole tooth; TCA = transverse crown apex; TCB = transverse crown base; TMC = transverse mid-crown. Extensive diagenetic recrystallization of PMU 28760–28763 prevented measurement.

Specimen	Section	Labial	Lingual	Mesial-Distal	
PMU 28757/1	LCA	1.9	1.6	-	-
PMU 28757/2	LCMB	2.9	3.1	-	-
PMU 28758/1	TCA	1.5	1.4	2.2	2.4
PMU 28758/3	TMC	1.9	1.8	2.7	3.2
PMU 28758/5	TCB	1.8	1.9	2.4	2.6
PMU 28759/1	TCA	1.1	1.1	1.2	1.4
PMU 28760/2	TCB	-	-	-	-
PMU 28761/1	LWT	-	-	-	-
PMU 28762/1	LWT	-	-	-	-
PMU 28763/2	LWT	-	-	-	-

All petrographic slides (PMU 28757/1–4, PMU 28758/1–5, PMU 28759/1–2, PMU 28760/1–3, PMU 28761/1–4, PMU 28762/1–2, PMU 28763/1–5) and accompanying image metadata produced by the described study are publically accessible through the PMU fossil collection and digital archives.

## Results

Microstructural examination of the thin (~120–150 μm) prismatic enamel layers revealed discrete dark-coloured laminations (Fig 6C and 6D). These might represent striae of Retzius, which deposit over variable timeframes during enamel formation in modern amniotes [42, 45]. The laminations also became more pronounced within the external enamel ridges (Fig 6D), perhaps in response to mechanical stress incurred during force loading across the curved lingual surface of the tooth in life.

Odontocyte lacunae and radially arrayed dentinal tubules were observed extending from the pulp cavity (Fig 6C and 6E). Longitudinal central tubules projected towards the crown apex (Fig 6F). Extensive microstructural degradation along the enamel-dentine junction (Fig 6E, 6G and 6H) resembled microborings attributed to post-depositional bacterial or fungal activity [38, 39, 46]. Some sections otherwise preserved an interglobular zone at the orthodentine periphery (Fig 6G and 6H), and the tooth roots (where visible) incorporated an external layer of cementum.

Discolouration from secondary minerals highlighted faint concentric staining up to ~50 μm in thickness, which was only visible in some transverse sections (Fig 6I). Occasionally this intercalated with widely-spaced dark and light coloured bands up to ~30 μm apart (Fig 6J) that could represent traces of long-period Andresen lines accumulated over multiple days [39, 40, 42]. Finer alternating layers 1.2–5.3 μm wide (Fig 6K; Table 2) corresponded with incremental markings elsewhere interpreted as daily lines of von Ebner [39, 40, 42]. We therefore adapted the approach of Gren and Lindgren [38] in multiplying the mean line thickness of 1.8–3.2 μm from the longitudinal midline section through PMU 28757/1–2, by the corresponding dentine thickness range of 1750–3000 μm measured at the apex and crown base, to estimate a total of 547–1667 periodic increments across the whole tooth (Table 4). This yielded an average dentine depositional rate of 950 days based on the cumulative mean of all measurement locations [38], and an extraordinarily prolonged tooth formation time of 2.6 years (full range = 1.5–4.5 years from the apex versus tooth base) following approximation of the Cretaceous year at 370.3–371 days [47, 48] (Table 4).

**Table 4:** Estimated dentine formation rates for selected PMU *Scanisaurus* teeth. Formation rate for PMU 28757/1–2 was averaged from cumulative measurements taken along the longitudinal midline of the whole tooth (LWT). *Cretaceous years [47, 48].

Specimen	Section	Dentine Width (μm)	Increment Width (μm)	Total Increments (Rate in days)	Formation Rate (Creta. years)*
PMU 28757/1	LCA	1750	3.2	547	1.5
PMU 28757/2	LCMB	3000	1.8	1667	4.5
PMU 28757/1–2	LWT	2375	2.5	950	2.6
PMU 28759/1	TCA	1200	2.8	429	1.2
PMU 28758/3	TMC	2400	1.9	1263	3.4

## Discussion

Our evidence for protracted tooth formation in dental remains attributed to the Late Cretaceous elasmosaurid *Scanisaurus* concurs with previous reports of extended dental replacement cycles in plesiosaurians and other more basal sauropterygians [18, 26–28]. Indeed, delayed replacement periodicity might have been broadly characteristic of these clades (and been influenced by thecodont gomphosis, indeterminate growth and metabolism [49]), but has been more specifically linked to regionalized tooth shape variation and enlargement in heterodont pliosaurids [18]. The detection of prolonged tooth formation times in *Scanisaurus* could therefore indicate a comparable adaptation towards discretized tooth function, which in anisodont elasmosaurids presumably involved selective elongation of the premaxillary-maxillary and dentary ‘fangs’ via modified growth periods and rate of odontoblast/ameloblast differentiation during apical dentine secretion [42]. Nevertheless, not all elasmosaurids were anisodont [13, 20, 21], implying that disparate tooth arrangements could evince contrasting formation rates between elasmosaurid taxa (a phenomenon recognized elsewhere in polyphyodont amniotes [37, 50]). This has particular pertinence for *Scanisaurus*, which is frequently compared with homodont elasmosaurids such as *Aristonectes* [31, 34], but may have alternative affinities based on its delayed tooth formation pattern.

Another important implication is the increased likelihood of severe tooth wear, breakage and/or related disease [30, 37]. Certainly, dental pathologies have been described in plesiosaurians before (mainly larger-skulled ‘pliosauromorphs’ [10, 51]), as has chronic tooth-associated bone deformation [52]. Notably, densely spaced dentine increments [39], apparently slow tooth replacement [53], and caries [54] have also all been documented in ichthyosaurians. In contrast, only superficial wear and missing teeth have thus far been described in elasmosaurids [6, 10], which likewise lacked robust crowns more typically associated with prolonged tooth development and macrophagous diets [10, 30, 38]. This might be explained by preferential feeding (inferred from bromalite contents [10, 14, 16]) on relatively small, easily subdued prey that was swallowed whole and processed using gastroliths in the gut [55]. Such dietary specialization potentially evolved hand-in-hand with the need to minimise excessive tooth damage, and was likely further coupled with selective foraging strategies that employed the slender interdigitating teeth to entrap, pierce or sieve prey from the water column and fine seafloor sediments (e.g. silt and mud); non-selective suspension feeding has been critiqued because of structural constraints on the reptilian pharynx [56]. Delayed tooth formation, together with ‘alveolarized’ replacement and heterogenous dental configurations could therefore have been a key factor constraining the adaptive radiation of elasmosaurids as middle trophic-level aquatic predators, and perhaps indirectly contributed to their environmental [57, 58] and geographical prevalence [19] via the capacity to utilize a wider range of available food resources.
